# Bis[μ-(*E*)-*N*′-(4-oxido-4-phenyl­but-3-en-2-yl­idene)benzohydrazidato]bis­[pyridine­copper(II)]

**DOI:** 10.1107/S1600536810019902

**Published:** 2010-06-05

**Authors:** Mehdi Hatefi, Iran Sheikhshoaie, Majid Moghadam, Valiollah Mirkhani, Reza Kia

**Affiliations:** a Chemistry Department, Shahid Bahonar University, Kerman, Iran; b Department of Chemistry, University of Isfahan, Isfahan 81746-73441, Iran; c Department of Chemistry, Science and Research Branch, Islamic Azad University, Tehran, Iran; d X-ray Crystallography Lab., Plasma Physics Research Center, Science and Research Branch, Islamic Azad University, Tehran, Iran

## Abstract

In the crystal structure of the title centrosymmetric dimer, [Cu_2_(C_17_H_14_N_2_O_2_)_2_(C_5_H_5_N)_2_], the Cu^II^ atom has an almost perfect square-pyramidal geometry. The Cu^II^ ion is coordin­ated by the NO_2_ donor atoms of the hydrazide Schiff base ligand, the N atom of the pyridine group and an O atom of the symmetry-related unit. The dihedral angles between the pyridine ring and the two phenyl rings of the ligand are 21.4 (3) and 24.0 (2)°. The mol­ecular structure is stabilized by intra­molecular C—H⋯O inter­actions.

## Related literature

For background to the properties of hydrazide Schiff base–metal complexes, see: Rao *et al.* (1990 ▶); West *et al.* (1993 ▶). For bond-length data, see: Allen *et al.* (1987 ▶).
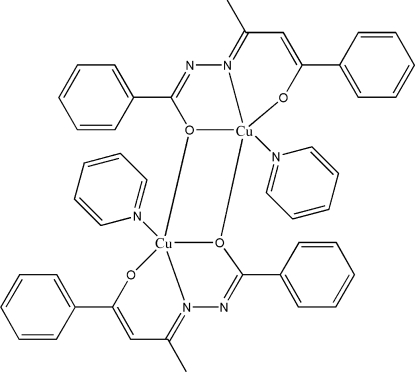


## Experimental

### 

#### Crystal data

[Cu_2_(C_17_H_14_N_2_O_2_)_2_(C_5_H_5_N)_2_]
*M* *_r_* = 841.88
Monoclinic, 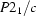

*a* = 9.2678 (19) Å
*b* = 20.903 (4) Å
*c* = 11.907 (4) Å
β = 122.65 (2)°
*V* = 1942.2 (8) Å^3^
*Z* = 2
Mo *K*α radiation
μ = 1.15 mm^−1^
*T* = 296 K
0.25 × 0.19 × 0.08 mm

#### Data collection

Stoe IPDS II diffractometer
Absorption correction: multi-scan (*MULABS* in *PLATON*; Blessing, 1995 ▶; Spek, 2009 ▶) *T*_min_ = 0.791, *T*_max_ = 1.179
22473 measured reflections
3419 independent reflections
2396 reflections with *I* > 2σ(*I*)
*R*_int_ = 0.086

#### Refinement

*R*[*F* ^2^ > 2σ(*F* ^2^)] = 0.048
*wR*(*F* ^2^) = 0.091
*S* = 0.98
3419 reflections
248 parameters
H-atom parameters constrained
Δρ_max_ = 0.60 e Å^−3^
Δρ_min_ = −0.24 e Å^−3^

### 

Data collection: *X-AREA* (Stoe & Cie, 2007 ▶); cell refinement: *X-AREA*; data reduction: *X-RED* (Stoe & Cie, 2007 ▶); program(s) used to solve structure: *SHELXS97* (Sheldrick, 2008 ▶); program(s) used to refine structure: *SHELXL97* (Sheldrick, 2008 ▶); molecular graphics: *SHELXTL* (Sheldrick, 2008 ▶); software used to prepare material for publication: *SHELXTL* and *PLATON* (Spek, 2009 ▶).

## Supplementary Material

Crystal structure: contains datablocks global, I. DOI: 10.1107/S1600536810019902/su2179sup1.cif

Structure factors: contains datablocks I. DOI: 10.1107/S1600536810019902/su2179Isup2.hkl

Additional supplementary materials:  crystallographic information; 3D view; checkCIF report

## Figures and Tables

**Table 1:** Hydrogen-bond geometry (Å, °)

*D*—H⋯*A*	*D*—H	H⋯*A*	*D*⋯*A*	*D*—H⋯*A*
C18—H18*A*⋯O1	0.93	2.34	2.892 (6)	117
C22—H22*A*⋯O2	0.93	2.37	2.940 (5)	119

**Fig. 1.**
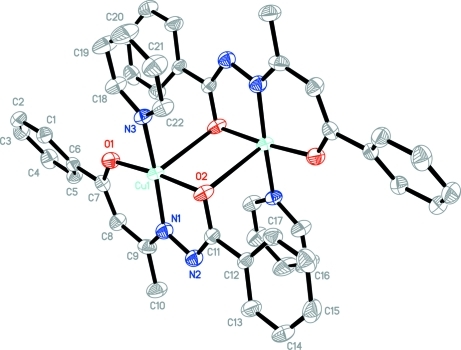
A view of the molecular structure of the title complex, showing 40% probability displacement ellipsoids (H-toms have been omitted for clarity).

**Fig. 2.**
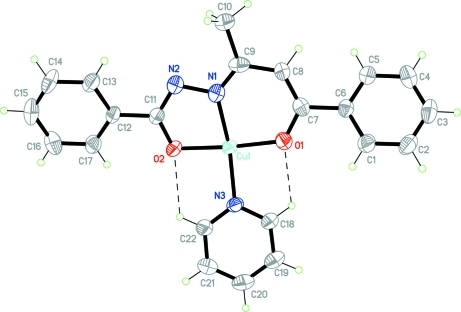
The asymmetric unit of the title compound. The dashed lines show the intramolecular C-H···O interactions.

**Fig. 3.**
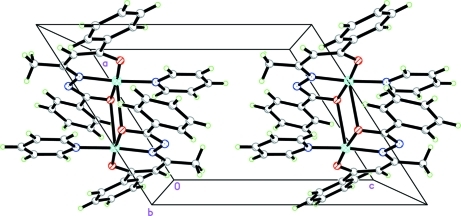
The crystal packing of the title compound, viewed down the b-axis.

## supplementary crystallographic information

### Comment

Among ligand systems hydrazines and hydrazones occupy a special place because the transition metal complexes of these ligands, developed due to their chelating capacity and structural flexibility, have interesting electrical as well as magnetic properties (Rao *et al.*, 1990), and pharmacological activities, such as antibacterial, antitumoural, antiviral antimalaria, antituberculosis (West *et al.*, 1993).

The molecular structure of the title molecule is illustrated in Fig. I. It is a novel hydrazido-Schiff base copper(II) complex, with the Cu^II^ atom having a N2O2 square-pyramidal geometry. The bond lengths (Allen *et al.*, 1987) and angles are within the normal ranges. The dihedral angles between the pyridine ring (N3/C18-C22) and the phenyl rings, A (C1-C6) and B (C12-C17), of the ligand are 21.4 (3) and 24.0 (2)°, respectively. The  molecular structure is stabilized by intramolecular C—H···O interactions (Fig. 2, Table 1).

In the crystal the molecules are held together by normal van der Waals interactions (Fig. 3).

### Experimental

The title compound was synthesized by adding (*E*)-*N'*- (4-oxo-4-phenyl butane-2-ylidene) benzohydrazide (1 mmol) to a solution of Cu(OAc)_2_. H_2_O (1 mmol) in methanol (30 ml). The mixture was refluxed with stirring for 30 min. It was then placed under a fume hood, near to the a solution of another sample which had pyridine as solvent of crystallization. As a consequence pyridine diffused into the methanol solution and resulted in the formation of brown single crystals of the title complex, over several days.

### Refinement

The H-atoms were positioned geometrically and refined using a riding model approximation: C-H = 0.96 Å for H-methyl and 0.93 Å for all other H-atoms, with U_iso_(H) = k × U_eq_(C), where k = 1.5 for H-methyl and = 1.2 for all other H-atoms.

### Crystal data

**Table:**

[Cu_2_(C_17_H_14_N_2_O_2_)_2_(C_5_H_5_N)_2_]	*F*(000) = 868
*M**_r_* = 841.88	*D*_x_ = 1.440 Mg m^−^^3^
Monoclinic, *P*2_1_/*c*	Mo *K*α radiation, λ = 0.71073 Å
Hall symbol: -P 2ybc	Cell parameters from 2500 reflections
*a* = 9.2678 (19) Å	θ = 2.3–27.8°
*b* = 20.903 (4) Å	µ = 1.15 mm^−^^1^
*c* = 11.907 (4) Å	*T* = 296 K
β = 122.65 (2)°	Block, brown
*V* = 1942.2 (8) Å^3^	0.25 × 0.19 × 0.08 mm
*Z* = 2	

### Data collection

**Table:**

Stoe IPDS II diffractometer	3419 independent reflections
Radiation source: fine-focus sealed tube	2396 reflections with *I* > 2σ(*I*)
graphite	*R*_int_ = 0.086
φ and ω scans	θ_max_ = 25.0°, θ_min_ = 2.0°
Absorption correction: multi-scan (*MULABS* in *PLATON*; Blessing, 1995; Spek, 2009)	*h* = −11→11
*T*_min_ = 0.791, *T*_max_ = 1.179	*k* = −24→23
22473 measured reflections	*l* = −14→14

### Refinement

**Table:**

Refinement on *F*^2^	Primary atom site location: structure-invariant direct methods
Least-squares matrix: full	Secondary atom site location: difference Fourier map
*R*[*F*^2^ > 2σ(*F*^2^)] = 0.048	Hydrogen site location: inferred from neighbouring sites
*wR*(*F*^2^) = 0.091	H-atom parameters constrained
*S* = 0.98	*w* = 1/[σ^2^(*F*_o_^2^) + (0.0421*P*)^2^] where *P* = (*F*_o_^2^ + 2*F*_c_^2^)/3
3419 reflections	(Δ/σ)_max_ < 0.001
248 parameters	Δρ_max_ = 0.60 e Å^−^^3^
0 restraints	Δρ_min_ = −0.24 e Å^−^^3^

### Special details

**Table:**

Geometry. All esds (except the esd in the dihedral angle between two l.s. planes) are estimated using the full covariance matrix. The cell esds are taken into account individually in the estimation of esds in distances, angles and torsion angles; correlations between esds in cell parameters are only used when they are defined by crystal symmetry. An approximate (isotropic) treatment of cell esds is used for estimating esds involving l.s. planes.
Refinement. Refinement of F^2^ against ALL reflections. The weighted R-factor wR and goodness of fit S are based on F^2^, conventional R-factors R are based on F, with F set to zero for negative F^2^. The threshold expression of F^2^ > 2sigma(F^2^) is used only for calculating R-factors(gt) etc. and is not relevant to the choice of reflections for refinement. R-factors based on F^2^ are statistically about twice as large as those based on F, and R- factors based on ALL data will be even larger.

### Fractional atomic coordinates and isotropic or equivalent isotropic displacement parameters (Å^2^)

**Table:**

	*x*	*y*	*z*	*U*_iso_*/*U*_eq_	
Cu1	0.72076 (6)	0.989490 (19)	0.59789 (4)	0.03829 (14)	
O1	0.8469 (3)	0.91348 (12)	0.6768 (2)	0.0487 (6)	
O2	0.5995 (3)	1.06734 (11)	0.5062 (2)	0.0483 (7)	
N1	0.7488 (4)	0.98572 (15)	0.4481 (3)	0.0472 (5)	
N2	0.6903 (4)	1.04093 (15)	0.3654 (3)	0.0472 (5)	
N3	0.7158 (4)	1.01066 (15)	0.7612 (3)	0.0414 (7)	
C1	1.0439 (5)	0.8085 (2)	0.8269 (4)	0.0516 (10)	
H1A	1.0469	0.8445	0.8738	0.062*	
C2	1.1150 (5)	0.7523 (2)	0.8941 (4)	0.0604 (12)	
H2A	1.1679	0.7510	0.9862	0.073*	
C3	1.1088 (6)	0.6981 (2)	0.8270 (5)	0.0641 (12)	
H3A	1.1566	0.6601	0.8730	0.077*	
C4	1.0307 (6)	0.7005 (2)	0.6901 (5)	0.0635 (12)	
H4A	1.0251	0.6637	0.6438	0.076*	
C5	0.9611 (5)	0.75679 (18)	0.6218 (4)	0.0517 (10)	
H5A	0.9094	0.7580	0.5297	0.062*	
C6	0.9678 (4)	0.81204 (17)	0.6902 (4)	0.0404 (9)	
C7	0.8931 (5)	0.87399 (17)	0.6172 (4)	0.0435 (9)	
C8	0.8818 (5)	0.88587 (18)	0.5004 (4)	0.0486 (10)	
H8A	0.9209	0.8535	0.4696	0.058*	
C9	0.8162 (5)	0.94287 (19)	0.4177 (4)	0.0509 (10)	
C10	0.8303 (6)	0.9466 (2)	0.2981 (4)	0.0630 (12)	
H10A	0.8992	0.9829	0.3068	0.095*	
H10B	0.7180	0.9512	0.2190	0.095*	
H10C	0.8826	0.9082	0.2921	0.095*	
C11	0.6189 (4)	1.07952 (16)	0.4061 (3)	0.0377 (8)	
C12	0.5563 (4)	1.14232 (16)	0.3377 (3)	0.0390 (8)	
C13	0.5943 (5)	1.16228 (19)	0.2453 (4)	0.0555 (11)	
H13A	0.6581	1.1360	0.2249	0.067*	
C14	0.5383 (6)	1.2207 (2)	0.1837 (5)	0.0695 (13)	
H14A	0.5662	1.2338	0.1231	0.083*	
C15	0.4425 (6)	1.2595 (2)	0.2104 (5)	0.0663 (13)	
H15A	0.4033	1.2985	0.1669	0.080*	
C16	0.4035 (5)	1.2407 (2)	0.3021 (5)	0.0625 (12)	
H16A	0.3388	1.2673	0.3211	0.075*	
C17	0.4606 (5)	1.18260 (17)	0.3654 (4)	0.0476 (10)	
H17A	0.4347	1.1702	0.4276	0.057*	
C18	0.7643 (6)	0.96766 (19)	0.8587 (4)	0.0561 (11)	
H18A	0.7996	0.9274	0.8495	0.067*	
C19	0.7642 (7)	0.9806 (3)	0.9721 (4)	0.0797 (15)	
H19A	0.8004	0.9498	1.0385	0.096*	
C20	0.7102 (7)	1.0389 (2)	0.9855 (5)	0.0814 (16)	
H20A	0.7094	1.0486	1.0614	0.098*	
C21	0.6573 (6)	1.0830 (2)	0.8863 (4)	0.0700 (13)	
H21A	0.6188	1.1230	0.8931	0.084*	
C22	0.6618 (5)	1.06729 (19)	0.7760 (4)	0.0514 (10)	
H22A	0.6256	1.0976	0.7087	0.062*	

### Atomic displacement parameters (Å^2^)

**Table:**

	*U*^11^	*U*^22^	*U*^33^	*U*^12^	*U*^13^	*U*^23^
Cu1	0.0494 (3)	0.0364 (2)	0.0337 (2)	−0.0004 (2)	0.02546 (19)	0.0017 (2)
O1	0.0575 (17)	0.0441 (15)	0.0517 (16)	0.0063 (13)	0.0342 (14)	0.0053 (12)
O2	0.0690 (18)	0.0422 (15)	0.0389 (15)	−0.0001 (13)	0.0324 (14)	0.0067 (11)
N1	0.0515 (13)	0.0442 (12)	0.0462 (13)	0.0004 (11)	0.0266 (12)	0.0062 (10)
N2	0.0515 (13)	0.0442 (12)	0.0462 (13)	0.0004 (11)	0.0266 (12)	0.0062 (10)
N3	0.0501 (17)	0.0420 (17)	0.0330 (15)	−0.0047 (15)	0.0231 (14)	−0.0017 (15)
C1	0.049 (2)	0.059 (3)	0.049 (2)	0.010 (2)	0.028 (2)	0.007 (2)
C2	0.057 (3)	0.079 (3)	0.047 (2)	0.015 (2)	0.029 (2)	0.021 (2)
C3	0.058 (3)	0.059 (3)	0.072 (3)	0.014 (2)	0.033 (3)	0.029 (3)
C4	0.064 (3)	0.045 (3)	0.077 (3)	0.009 (2)	0.035 (3)	0.003 (2)
C5	0.056 (3)	0.049 (3)	0.048 (2)	0.008 (2)	0.027 (2)	0.007 (2)
C6	0.034 (2)	0.044 (2)	0.044 (2)	0.0040 (17)	0.0213 (18)	0.0074 (17)
C7	0.038 (2)	0.041 (2)	0.052 (2)	−0.0006 (17)	0.0237 (19)	−0.0020 (18)
C8	0.059 (3)	0.039 (2)	0.047 (2)	0.0087 (19)	0.028 (2)	0.0037 (18)
C9	0.056 (3)	0.061 (3)	0.047 (2)	−0.007 (2)	0.036 (2)	−0.008 (2)
C10	0.087 (3)	0.064 (3)	0.058 (3)	0.016 (2)	0.053 (3)	0.012 (2)
C11	0.038 (2)	0.035 (2)	0.0330 (19)	−0.0079 (16)	0.0148 (17)	−0.0002 (15)
C12	0.040 (2)	0.036 (2)	0.0319 (19)	−0.0064 (16)	0.0139 (17)	0.0040 (16)
C13	0.068 (3)	0.054 (3)	0.054 (3)	0.005 (2)	0.039 (2)	0.014 (2)
C14	0.081 (3)	0.066 (3)	0.068 (3)	0.001 (3)	0.044 (3)	0.031 (2)
C15	0.056 (3)	0.047 (3)	0.074 (3)	0.001 (2)	0.020 (3)	0.022 (2)
C16	0.054 (3)	0.045 (3)	0.087 (3)	0.004 (2)	0.036 (3)	0.002 (2)
C17	0.051 (2)	0.043 (2)	0.051 (2)	−0.0061 (19)	0.029 (2)	0.0037 (18)
C18	0.080 (3)	0.049 (2)	0.045 (2)	0.004 (2)	0.038 (2)	0.0058 (18)
C19	0.126 (4)	0.078 (3)	0.051 (3)	0.012 (3)	0.058 (3)	0.013 (3)
C20	0.127 (5)	0.082 (3)	0.052 (3)	0.007 (3)	0.059 (3)	−0.002 (3)
C21	0.102 (4)	0.058 (3)	0.058 (3)	0.006 (3)	0.048 (3)	−0.009 (2)
C22	0.072 (3)	0.041 (2)	0.046 (2)	−0.003 (2)	0.034 (2)	−0.0011 (18)

### Geometric parameters (Å, °)

**Table:**

Cu1—O1	1.894 (2)	C9—C10	1.500 (5)
Cu1—N1	1.938 (3)	C10—H10A	0.9600
Cu1—O2	1.944 (2)	C10—H10B	0.9600
Cu1—N3	2.018 (3)	C10—H10C	0.9600
O1—C7	1.301 (4)	C11—C12	1.488 (5)
O2—C11	1.323 (4)	C12—C17	1.385 (5)
N1—C9	1.252 (5)	C12—C13	1.389 (5)
N1—N2	1.421 (4)	C13—C14	1.373 (6)
N2—C11	1.292 (4)	C13—H13A	0.9300
N3—C22	1.333 (5)	C14—C15	1.361 (6)
N3—C18	1.340 (4)	C14—H14A	0.9300
C1—C2	1.372 (5)	C15—C16	1.379 (6)
C1—C6	1.383 (5)	C15—H15A	0.9300
C1—H1A	0.9300	C16—C17	1.376 (5)
C2—C3	1.370 (6)	C16—H16A	0.9300
C2—H2A	0.9300	C17—H17A	0.9300
C3—C4	1.381 (6)	C18—C19	1.377 (6)
C3—H3A	0.9300	C18—H18A	0.9300
C4—C5	1.378 (5)	C19—C20	1.359 (6)
C4—H4A	0.9300	C19—H19A	0.9300
C5—C6	1.395 (5)	C20—C21	1.365 (6)
C5—H5A	0.9300	C20—H20A	0.9300
C6—C7	1.504 (5)	C21—C22	1.376 (5)
C7—C8	1.361 (5)	C21—H21A	0.9300
C8—C9	1.453 (5)	C22—H22A	0.9300
C8—H8A	0.9300		
			
O1—Cu1—N1	93.94 (12)	C9—C10—H10A	109.5
O1—Cu1—O2	174.68 (10)	C9—C10—H10B	109.5
N1—Cu1—O2	80.87 (12)	H10A—C10—H10B	109.5
O1—Cu1—N3	92.01 (12)	C9—C10—H10C	109.5
N1—Cu1—N3	168.38 (13)	H10A—C10—H10C	109.5
O2—Cu1—N3	92.90 (11)	H10B—C10—H10C	109.5
C7—O1—Cu1	123.9 (2)	N2—C11—O2	124.3 (3)
C11—O2—Cu1	110.4 (2)	N2—C11—C12	118.2 (3)
C9—N1—N2	116.4 (3)	O2—C11—C12	117.4 (3)
C9—N1—Cu1	129.1 (3)	C17—C12—C13	118.3 (3)
N2—N1—Cu1	114.5 (2)	C17—C12—C11	121.1 (3)
C11—N2—N1	109.2 (3)	C13—C12—C11	120.6 (3)
C22—N3—C18	117.1 (3)	C14—C13—C12	120.4 (4)
C22—N3—Cu1	121.8 (2)	C14—C13—H13A	119.8
C18—N3—Cu1	121.1 (3)	C12—C13—H13A	119.8
C2—C1—C6	120.8 (4)	C15—C14—C13	120.8 (4)
C2—C1—H1A	119.6	C15—C14—H14A	119.6
C6—C1—H1A	119.6	C13—C14—H14A	119.6
C3—C2—C1	120.8 (4)	C14—C15—C16	119.9 (4)
C3—C2—H2A	119.6	C14—C15—H15A	120.1
C1—C2—H2A	119.6	C16—C15—H15A	120.1
C2—C3—C4	119.3 (4)	C17—C16—C15	119.8 (4)
C2—C3—H3A	120.4	C17—C16—H16A	120.1
C4—C3—H3A	120.4	C15—C16—H16A	120.1
C5—C4—C3	120.5 (4)	C16—C17—C12	120.9 (4)
C5—C4—H4A	119.7	C16—C17—H17A	119.6
C3—C4—H4A	119.7	C12—C17—H17A	119.6
C4—C5—C6	120.2 (4)	N3—C18—C19	122.7 (4)
C4—C5—H5A	119.9	N3—C18—H18A	118.6
C6—C5—H5A	119.9	C19—C18—H18A	118.6
C1—C6—C5	118.4 (3)	C20—C19—C18	119.1 (4)
C1—C6—C7	120.6 (3)	C20—C19—H19A	120.4
C5—C6—C7	120.9 (3)	C18—C19—H19A	120.4
O1—C7—C8	125.1 (3)	C19—C20—C21	119.1 (4)
O1—C7—C6	114.6 (3)	C19—C20—H20A	120.4
C8—C7—C6	120.3 (3)	C21—C20—H20A	120.4
C7—C8—C9	128.0 (3)	C20—C21—C22	118.9 (4)
C7—C8—H8A	116.0	C20—C21—H21A	120.5
C9—C8—H8A	116.0	C22—C21—H21A	120.5
N1—C9—C8	118.9 (3)	N3—C22—C21	123.0 (4)
N1—C9—C10	123.3 (4)	N3—C22—H22A	118.5
C8—C9—C10	117.8 (3)	C21—C22—H22A	118.5
			
N1—Cu1—O1—C7	−11.1 (3)	O1—C7—C8—C9	−0.8 (7)
N3—Cu1—O1—C7	178.8 (3)	C6—C7—C8—C9	−179.0 (4)
N1—Cu1—O2—C11	−7.6 (2)	N2—N1—C9—C8	178.7 (3)
N3—Cu1—O2—C11	162.6 (2)	Cu1—N1—C9—C8	1.6 (6)
O1—Cu1—N1—C9	5.3 (4)	N2—N1—C9—C10	−2.4 (6)
O2—Cu1—N1—C9	−175.9 (4)	Cu1—N1—C9—C10	−179.5 (3)
N3—Cu1—N1—C9	125.9 (6)	C7—C8—C9—N1	−5.9 (7)
O1—Cu1—N1—N2	−171.9 (2)	C7—C8—C9—C10	175.2 (4)
O2—Cu1—N1—N2	6.9 (2)	N1—N2—C11—O2	−2.3 (5)
N3—Cu1—N1—N2	−51.3 (7)	N1—N2—C11—C12	176.8 (3)
C9—N1—N2—C11	177.7 (3)	Cu1—O2—C11—N2	8.0 (4)
Cu1—N1—N2—C11	−4.7 (4)	Cu1—O2—C11—C12	−171.2 (2)
O1—Cu1—N3—C22	169.8 (3)	N2—C11—C12—C17	172.2 (3)
N1—Cu1—N3—C22	49.0 (8)	O2—C11—C12—C17	−8.7 (5)
O2—Cu1—N3—C22	−8.2 (3)	N2—C11—C12—C13	−8.5 (5)
O1—Cu1—N3—C18	−11.3 (3)	O2—C11—C12—C13	170.6 (3)
N1—Cu1—N3—C18	−132.1 (6)	C17—C12—C13—C14	0.2 (6)
O2—Cu1—N3—C18	170.7 (3)	C11—C12—C13—C14	−179.1 (4)
C6—C1—C2—C3	−1.5 (6)	C12—C13—C14—C15	−1.1 (7)
C1—C2—C3—C4	0.4 (7)	C13—C14—C15—C16	1.2 (7)
C2—C3—C4—C5	0.6 (7)	C14—C15—C16—C17	−0.5 (7)
C3—C4—C5—C6	−0.4 (6)	C15—C16—C17—C12	−0.4 (6)
C2—C1—C6—C5	1.7 (6)	C13—C12—C17—C16	0.5 (6)
C2—C1—C6—C7	−178.8 (4)	C11—C12—C17—C16	179.8 (3)
C4—C5—C6—C1	−0.7 (6)	C22—N3—C18—C19	−1.7 (6)
C4—C5—C6—C7	179.8 (4)	Cu1—N3—C18—C19	179.4 (4)
Cu1—O1—C7—C8	10.8 (5)	N3—C18—C19—C20	1.0 (8)
Cu1—O1—C7—C6	−170.9 (2)	C18—C19—C20—C21	0.2 (8)
C1—C6—C7—O1	−24.9 (5)	C19—C20—C21—C22	−0.7 (8)
C5—C6—C7—O1	154.6 (3)	C18—N3—C22—C21	1.1 (6)
C1—C6—C7—C8	153.5 (4)	Cu1—N3—C22—C21	−179.9 (3)
C5—C6—C7—C8	−27.0 (5)	C20—C21—C22—N3	0.0 (7)

### Hydrogen-bond geometry (Å, °)

**Table:**

*D*—H···*A*	*D*—H	H···*A*	*D*···*A*	*D*—H···*A*
C18—H18A···O1	0.93	2.34	2.892 (6)	117
C22—H22A···O2	0.93	2.37	2.940 (5)	119
